# ﻿A contribution to the genus *Didrepanephorus* Wood-Mason, 1878 (Coleoptera, Scarabaeidae, Rutelinae)

**DOI:** 10.3897/zookeys.1092.75831

**Published:** 2022-04-04

**Authors:** Ming-Zhi Zhao, Wei-Xin Liu

**Affiliations:** 1 College of Plant Protection, South China Agricultural University, Guangzhou, 510642, China South China Agricultural University Guangzhou China

**Keywords:** China, Didrepanephorina, lectotype, Myanmar, new combination, new record, new species, Rutelini, Scarabaeoidea

## Abstract

The diagnostic characters of the genera *Didrepanephorus* Wood-Mason, 1878 and *Fruhstorferia* Kolbe, 1894 are clarified. The following nomenclatorial acts are proposed: *Didrepanephorusbirmanicus* (Arrow, 1907), **comb. nov.**, *Didrepanephorusfukinukii* (Muramoto & Araya, 2000), **comb. nov.**, *Fruhstorferiabaron* (Prokofiev, 2013), **comb. nov.**, and *Fruhstorferiaanthracina* Ohaus, 1903, **comb. rev.***Didrepanephorustangzhaoyangi* Zhao & Liu, **sp. nov.** is described from Yunnan Province, China. A lectotype is designated for *Fruhstorferiabirmanica* Arrow, 1907. *Didrepanephorusmizunumai* Nagai & Hirasawa, 1991 is reported from Myanmar for the first time.

## ﻿Introduction

The subtribe Didrepanephorina was established by Ohaus (1918) to accommodate a morphologically remarkable genus *Didrepanephorus* Wood-Mason, 1878. The type species of this genus, *D.bifalcifer* Wood-Mason, 1878, is a densely setose ruteline beetle with strong sexual dimorphism in the shape of mandibles (Wood-Mason 1878). The morphological affinities between *Didrepanephorus* and *Fruhstorferia* Kolbe, 1894 had resulted in a chaos of generic separations when different characters were referred: Arrow (1917) separated *Fruhstorferia* from *Didrepanephorus* mainly by the dorsal surface generally without dense setae. Ohaus (1934a) recognized *Didrepanephorus*species by the complete frontoclypeal suture and reassigned species of the two genera. Young (1999) took the orientation of mandibles into account, i.e., bent upward in *Didrepanephorus* but directed forward in *Fruhstorferia*. Most recently, Muramoto (2005) differentiated the two genera by the prosternal process and abbreviation of abdominal ventrites 1–4 in males. Besides, some authors questioned the separation of the two genera (Nagai and Hirasawa 1991; Qiu et al. 2021). The authors of the present paper had found reliable morphological characters to distinguish the two genera. Based on these characters, the delimitation of the two genera is herein clarified.

The Chinese *Didrepanephorus*species are still poorly known. Qiu et al. (2021) described a new species from Guizhou and recorded two species new to Chinese fauna. Their biology was also reported, which greatly improved the knowledge of this genus. During the taxonomic study of the Old World Rutelini, a new species similar to *D.ohbayashii* (Nagai, 2004) was received by the first author from Yunnan Province of China. Additionally, based on the examination of the type material of *F.birmanica* Arrow, 1907, some taxonomic problems are also solved herein.

## ﻿Material and methods

Images of the external characters and male genitalia were taken using a Canon EOS 760D camera in conjunction with a Tamron 90 mm f/2.8 1:1 Macro Lens and a Laowa 25 mm f/2.8 2.5-5X Ultra Macro Lens, respectively. Zerene Stacker (version 1.04) was used for stacking. All images were modified and arranged in plates in Adobe Photoshop CS5.

Data of material in NHMUK are cited verbatim. Different labels are separated by a double slash (//). Specimens studied in this research are deposited in the following public and private collections:

**CCPC** Chang-Chin Chen’s personal collection, Tianjing, China;

**GGPC** Guy Guerlach’s personal collection, Orny, France;

**LCPC** Chao Li’s personal collection, Beijing, China;

**LZPC** Ze-Chuan Li’s personal collection, Tai’an, China;

**MYNU** Invertebrate Collection of Mianyang Normal University, Mianyang, China;

**NHMUK**The Natural History Museum, London, United Kingdom;

**SCAU**South China Agricultural University, Guangzhou, China;

**SFPC** Feng-Yi Sun’s personal collection, Yong’an, China;

**TZPC** Zhao-Yang Tang’s personal collection, Shenzhen, China;

**ZMPC** Ming-Zhi Zhao’s personal collection, Zhuhai, China.

## ﻿Taxonomy

### ﻿Delimitation of the genera *Didrepanephorus* Wood-Mason, 1878 and *Fruhstorferia* Kolbe, 1894

The following specimens were studied for comparative purpose: *D.yunnanuswakaharai* (Nagai, 2004): 1♂ (ZMPC), Laos, Houaphanh Province, Mt. Phu Pane, 2060 m, 2017.IV; *D.takuyai* (Muramoto, 2003): 1♂, 1♀ (ZMPC), Vietnam, Dalat, Lamdong, 2016.V; *Didrepanephorus* specimens studied in Qiu et al. (2021); *F.anthracina* Ohaus, 1903: 1♂, (CCPC), Vietnam, Bac Giang, Son Dong, Thanh Son, Tay Yen Tu, 120 m, 2014.VI.2, N.-Y. Tsai leg.; *F.javanajavana* Kolbe, 1894: 1♂, 1♀ (TZPC), Indonesia, West Java, 2016.VI; *F.nigromuliebris* Nagai, 1984: 1♂ (ZMPC), Sabah, Croker Range, 800 m, 2019.V; *Kibakoganeasexmaculata* (Kraatz, 1900): 3♂ (ZMPC), Vietnam, Yen Bai Province, Nghia Lo, 2017.VII; *K.akikoaesatoi* Nagai, 2004: 1♂ (ZMPC), Laos, Houaphanh Province, Mt. Phu Pane, 2060 m, 2016.IV; *K.yoshitomii* Nagai, 2004: 1♂ (ZMPC), Laos, Houaphanh Province, Mt. Phu Pane, 2060 m, 2016.III; *Masumotokoganeakinabalensis* (Ohaus, 1932): 1♂ (CCPC), Malaysia, Sabah, Tambunan, 1600 m, 2014.V.7, light, Yu-Tang Wang leg.

The authors of the present paper tentatively delimitate the two genera based on the shape of the male mandible and the range of body length. Regarding the *Didrepanephorus*species, there is a prominent protrusion at the base of lower margin of male mandible, which is either sharp or blunt at apex, and the lower margin is concave or strongly concave before the protrusion. Such prominent protrusion is replaced by a weak lump in *Fruhstorferia*, with exception of *F.flavipennis* Nagai, 1984, in which the lump is acute at apex. But the lower margin is never concave before the protrusion in *Fruhstorferia*. Sometimes there is a basal protrusion also at upper margin of mandible in *Didrepanephorus*species, which is absent in all *Fruhstorferia*species. The mandible of *Didrepanephorus* bent upwards, but directed forward in *Fruhstorferia*. The body length of *Didrepanephorus* varies from 13.0–22.4 mm in males (mandibles excluding) and 13.5–23.2 mm in females (Nagai and Hirasawa 1991; Muramoto and Araya 2000; Muramoto 2003b, 2005, 2013; Qiu et al. 2021). In the genus *Fruhstorferia*, the range is 22.0–31.1 mm in males (mandibles excluding) and 23.0–30.0 mm in females (Nagai 1984, 1989; Ohaus 1903; Prokofiev 2013). The range of body length is almost not overlapped and the genus *Fruhstorferia* is generally longer. At this stage, authors are unable to provide diagnostic characters of females due to lack of material.

The following nomenclatorial acts can be proposed based on the above delimitation: *D.birmanicus* (Arrow, 1907), comb. nov., *D.fukinukii* (Muramoto & Araya, 2000), comb. nov., *F.baron* (Prokofiev, 2013), comb. nov. and *F.anthracina* Ohaus, 1903, comb. rev. Prokofiev (2013) transferred *F.anthracina* to the genus *Didrepanephorus* because of the strongly abbreviated abdominal ventrites 1–4 in male. In the same paper, Prokofiev described a new species, i.e., *D.baron* and assigned it to the genus *Didrepanephorus* for the same reason. The two species lack basal protrusion at the lower margin of male mandible and have body longer, therefore they should be placed in the genus *Fruhstorferia*. The placement of *D.takuyai* (Muramoto, 2003) is still unclear: the male of this species has upright mandible without protrusion or lump at base of lower margin, and the body length of male can reach 23.5 mm (Muramoto 2003a). Consequently, a checklist of the two genera is provided (Table 1). Only type localities are cited to avoid probable misidentifications.

**Table 1.** Checklist of the genera *Didrepanephorus* and *Fruhstorferia* with type localities.

No.	Taxon name	Type locality (verbatim from original description)
1	*Didrepanephorusarnaudi* Muramoto, 2003	Bao Loc, 1400 m, Lam Dong Prov., Vietnam
2	*Didrepanephorusbifalcifer* Wood-Mason, 1878	Wakidgaon, a Village 30–35 miles S.E. of Sadia, in the valley of the Noa Dehing, India
3	*Didrepanephorusbirmanicus* (Arrow, 1907), **comb. nov.**	Ruby Mines, Burma
4	*Didrepanephorusfukinukii* (Muramoto & Araya, 2000), **comb. nov.**	Fang, Chiang-Mai Prov., N. Thailand
5	*Didrepanephorusheterocolor* Qiu, Zhao & Xu, 2021	Loudousenlin, 679 m, 25°17'51"N, 108°04'28"E, Maolan Nature Reserve, Libo County, Guizhou, China
6	*Didrepanephoruslamdongensis* Muramoto, 2013	Bao Loc, Lam Dong, South Vietnam
7	*Didrepanephoruslao* Nagai, 2005	Mt. Phu Pan, 1700 m, Ban Saleui, Xam Neua, Houa Phan Prov., N.E. Laos
8	*Didrepanephorusmizunumai* Nagai & Hirasawa, 1991	Fang, Chiangmai Prov., North Thailand
9	*Didrepanephorusmucronatus* Arrow, 1921	Laos, Indo-China
10	*Didrepanephorusnishiyamai* Muramoto, 2006	Mts. Damingshan, Guangxi Prov., China
11	*Didrepanephorusohbayashii* (Nagai, 2004) = *Didrepanephoruspilosus* Bouchard, 2007 (synonymized by Prokofiev 2014)	Ban Saleui, 1400 m, Xam Neua, Houa Phan Prov., N.E. Laos (*D.pilosus*: Mout Phouu-phien-kha-sieng, Dakchung District, Xekong, Laos)
12	*Didrepanephorussubvittatus* Benderitter, 1922	Chapa, Tonkin
13	*Didrepanephorustangzhaoyangi* Zhao & Liu, **sp. nov.**	Mangyun Village, 780 m, Taiping Town, Yingjiang County, Dehong Prefecture, Yunnan Prov., China
14	*Didrepanephorusvietnamicus* Muramoto & Kobayashi, 2019	Mt. Axan, 1300 m, Tay Giang, Quang Nam, Vietnam
15	*Didrepanephorusyunnanusyunnanus* (Ohaus, 1911)	Yunnan, China
16	*Didrepanephorusyunnanusclermonti* (Benderitter, 1929)	Chapa, Tonkin
17	*Didrepanephorusyunnanuskachinensis* Muramoto, 2005	Eastern Kachin State, Myanmar
18	*Didrepanephorusyunnanuspiaoacensis* (Nagai, 2004)	Mt. Pia Oac, Cao Bang Prov., N. Vietnam
19	*Didrepanephorusyunnanuswakaharai* (Nagai, 2004)	Mt. Phu Pan, 1750 m, Ban Saleui, Xam Neua, Houa Phan Prov., N.E. Laos
20	*Didrepanephoruszen* Muramoto, 2009	Pu Mat, Nghe An Prov., Vietnam
21	*Didrepanephorustakuyai* (Muramoto, 2003), **incertae sedis**	Mt. Braian, 45 km east of Bao Loc, Lam Dong Prov., Vietnam
22	*Fruhstorferiaanthracina* Ohaus, 1903, **comb. rev.**	Mauson Berge, Tonkin
23	*Fruhstorferiabaron* (Prokofiev, 2013), **comb. nov.**	about 72 km east of Dalat, 750–800 m, Khan Vinh County, KhanhHoa Prov. bordering Lamdong Prov., Dalat Plateau, Vietnam
24	*Fruhstorferiaegregia* Pouillaude, 1915	Kon-Tum, Annam
25	*Fruhstorferiaflavipennis* Nagai, 1984	Cameron Highlands, Pahang, Malaysia
26	*Fruhstorferiajavanajavana* Kolbe, 1894	West-Java
27	*Fruhstorferiajavanacastanea* Pouillaude, 1915	Monts Kawie, Java
28	*Fruhstorferianigromuliebris* Nagai, 1984	Croker range, ca. 1400 m, near Keningau city, Sabah, Malaysia
29	*Fruhstorferiaohtanii* Nagai, 1989	Lampung, South Sumatra, Indonesia

**Morphological remarks.** Minute and dense setae on dorsal surface can be traced in the species with glabrous appearance in *Didrepanephorus* and *Fruhstorferia*, as well as in the related genera *Kibakoganea* Nagai, 1984, *Masumotokoganea* Hirasawa, 1992 (the genera *Nagainokoganea* Hirasawa, 1992 and *Pukupuku* Muramoto, 2006 are not examined). This character sometimes has a different arrangement in closely allied species, e.g., *D.mizunumai* Nagai & Hirasawa, 1991 and *D.zen* Muramoto, 2009 (Muramoto 2009). It should be a synapomorphy shared by these genera. The complete frontoclypeal suture in *D.bifalcifer* (Ohaus 1934a) should be an autapomorphy, since its closest congener *D.mucronatus* Arrow, 1921 and other examined *Didrepanephorus*species have incomplete frontoclypeal suture (Qiu et al. 2021). And the prosternal process and medially abbreviated abdominal ventrites 1–4 in males (Muramoto 2005) are present in the species of both genera. It cannot be applied to distinguish the two genera.

#### ﻿ Didrepanephorus tangzhaoyangi

Taxon classificationAnimaliaColeopteraScarabaeidae

Zhao & Liu sp. nov.

0C70B02F-C579-5470-9889-57C304FC7AD6

http://zoobank.org/F7D27A21-A9C0-4141-A463-2471C3435350

Figs 1–6 , 7 , 9 , 10

##### Type locality.

China, Yunnan Province, Dehong Prefecture, Yingjiang County, Taiping Town, Mangyun Village, 780 m.

##### Type material

**(28 specimens). *Holotype*.** ♂ (SCAU), China: Yunnan Prov., Dehong Pref., Yingjiang County, Taiping Town, Mangyun, 780 m, 2021.VI.3, Shao-Fu Chen leg. // HOLOTYPE *Didrepanephorustangzhaoyangi* sp. nov. des. Zhao Ming-Zhi & Wei-Xin Liu 2021 [red label].

***Paratypes*** (14♂ & 13♀). 3♂, 2♀ (ZMPC), 1♂ (LZPC), same data as holotype; 2♂, 3♀ (TZPC), 1♀ (SCAU), 1♀ (ZMPC), same as preceding but 2021.VI.22; 2♂ & 1♀ (TZPC), same as preceding but 2020.VI; 1♂, 1♀ (MYNU), same as preceding but 750 m, 2019.V, local collector leg.; 1♂ (LCPC), CHINA: Yunnan Prov., Yingjiang County, Mangyun Village, 2017.VI.10; 2♂, 4♀ (TZPC), 1♂ (SFPC), China: Yunnan Prov., Dehong Pref., Yingjiang County, Taiping Town, Mangyun, Husonghe River, 750 m, 2021.VI, Zhao-Wei Guo leg.; 1♂ (CCPC), China: Yunnan Prov., Ruili Botanical Garden, 2013.V.6, Xiao-Dong Yang leg. All paratypes were provided with an additional yellow label: PARATYPE *Didrepanephorustangzhaoyangi* sp. nov. des. Zhao & Liu 2021.

##### Description of the holotype

**Figures 1–6.**
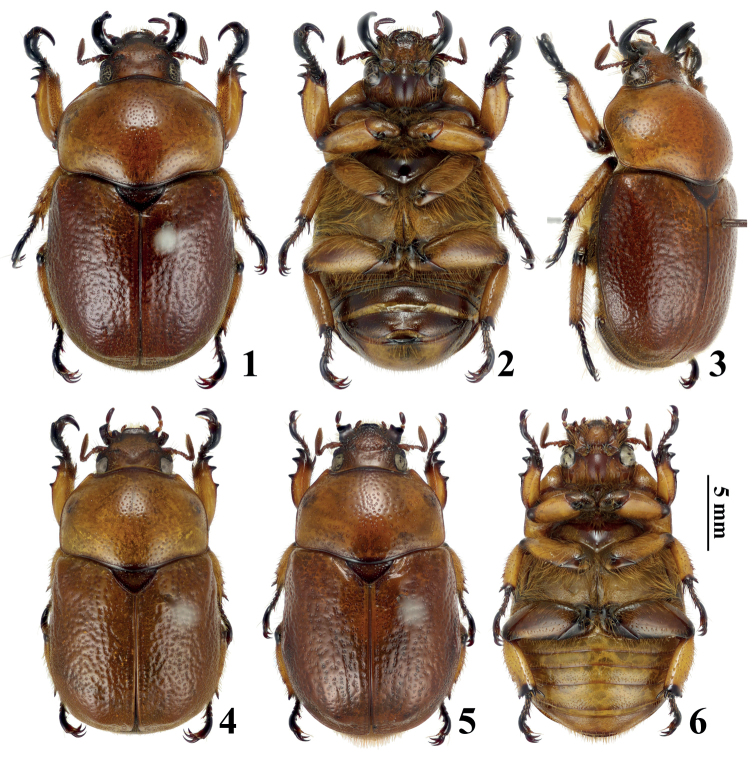
Habitus of *Didrepanephorustangzhaoyangi* Zhao, sp. nov. **1** holotype, dorsal view **2** holotype, ventral view **3** holotype, oblique lateral view **4** male paratype, dorsal view **5** female paratype, dorsal view **6** female paratype, ventral view.

**Figures 7–10.**
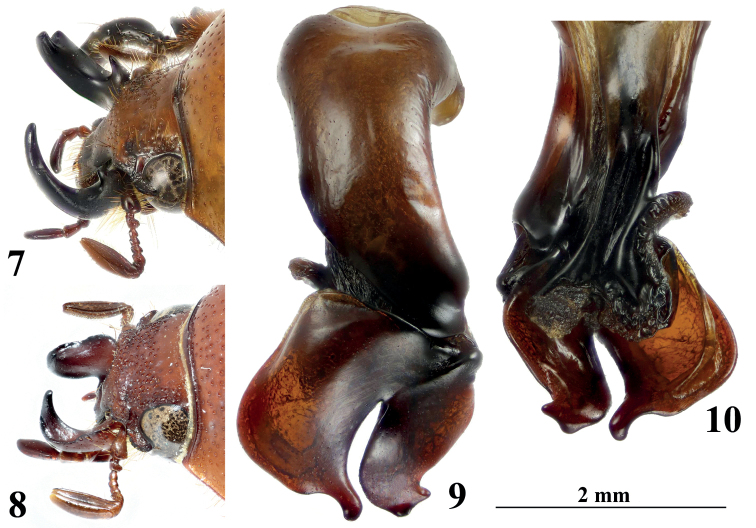
Morphological details of *Didrepanephorus*species**7** head of holotype of *D.tangzhaoyangi* Zhao & Liu, sp. nov., oblique lateral view **8** head of *D.birmanicus* (Arrow, 1907) (Lectotype of *Fruhstorferiabirmanica* Arrow, 1907),oblique lateral view, photo © Keita Matsumoto (NHMUK) **9–10** male genitalia of holotype of *D.tangzhaoyangi***9** dorsal view **10** ventral view. Scale bar for genitalia only.

(Figs 1–3, 7, 9–10). ***General*.** Body broadly ovoid and strongly convex. All external setae yellowish brown.

***Head*.** Dorsal surface yellowish brown, marginal portions darkened. Clypeus flat, trapezoidal, anterior margin nearly straight, anterior corner obtusely right-angled, side strongly convergent anteriad and weakly swollen in basal two fifth, then roundly curved, and almost subparallel in apical half; punctures large at disc but absent in middle, punctures small at marginal portions. Frontal-clypeal suture broadly interrupted medially, black at each side. Frons and vertex with scattered large punctures. Eyes canthus with roundly curved outer margin, not extends beyond outermost point of eye. Antenna reddish brown, length of antennal club distinctly shorter than antennomeres 2–7 combined. Dorsal surface of head with moderately dense, erect long setae, broadly glabrous medially. Labrum blackish brown, strongly exposed dorsally, with dense, erect long setae along margin, anterior margin feebly sinuate. Mandible blackish brown, bends upward, upper margin with a large, acute basal protrusion and a small, blunt proximal protrusion, lower margin with a large protrusion at base. Maxilla and maxillary palp reddish brown, maxilla with dense and long setae. Mentum yellowish brown, apical fourth of mentum darkened and slightly swollen, anterior margin curved but strongly concave medially, surface with sparse short setae each emerging from a puncture.

***Pronotum*.** Orange-brown, darkened at anterior and posterior margins. Strongly convex, ca. 1.46 × as wide as long, widest near middle. Anterior margin bisinuate; anterior marginal membrane complete. Sides feebly convergent posteriad in posterior half, roundly and broadly curved at middle, then strongly convergent anteriad in anterior half. Posterior margin broadly protruding in middle. All marginal lines complete. Anterior angle weakly protruding, posterior angle round. Surface with sparse large punctures, somewhat aggregate and smaller at disc. Each dorsal puncture accommodates a minute seta; lateral margin with a row of erect short setae.

***Scutellum*.** Brown, margin blackish brown. Semicircular in shape. Surface with sparse punctures, impunctate medially.

***Elytra*.** Dark orange-brown. Convex, length of each elytron slightly longer than cross width of the two elytra. Surface uneven, humeral and apical umbones weakly prominent. Strial punctures large and deep, punctures on primary striae as large as punctures on secondary striae, 1^st^ primary stria well defined by a longitudinal row of regular punctures, other striae unrecognizable; the whole surface with sparse small punctures, denser on lateral portions. Surface with moderately dense, semierect short setae, denser apically. Epipleura with a row of dense, short to moderately long setae.

**Propygidium and pygidium.** Brown, disc and apical portions of pygidium yellowish brown. Surfaces with moderately dense small punctures and erect, short to moderately long setae. Pygidium strongly convex and curved to ventrum, apex with a short row of erect long setae.

***Ventral thoracic surface*.** Ventral prothoracic surface yellowish brown, dark brown around procoxal cavity; surface with moderately dense, erect long setae, each emerging from a small puncture; anterior margin with a row of dense and very long setae. Prosternal process gradually narrower toward apex, with very dense, erect long setae. Ventral mesothoracic surface dark brown, with scattered small punctures; a broad middle portion smooth and glabrous, with an oblique row of small punctures at each side; each puncture with a recumbent short seta. Ventral metathoracic surface yellowish brown, gradually darkened toward each side; with dense small punctures and very long setae, less setose medially.

***Abdominal ventrites*.** Dark brown, ventrites 2–4 yellowish brown between anterior and posterior margin. Ventrites 1–4 strongly abbreviated; ventrite 6 strongly concave apically. Surface with the following arrangements of small punctures: moderately dense at medial portions of each ventrites, moderately dense and forming a row at each side near posterior margin of ventrites 1–5, sparse at each side of ventrites 1–5. Each puncture with a semierect short seta, which become moderately long to long at marginal portions and sides.

***Fore legs*.** Yellowish brown; joints of trochanter, femur, and tibia, including protibial spur and teeth dark brown; protarsus and claws blackish brown. Protibia tridentate, apical and proximal teeth sharp at apices; third tooth shorter and blunter, more spaced from the two anterior teeth. Protarsus strongly thickened, the weakly protruding ventrolateral apex of protarsomere 4 with a small spiniform seta at each side, protarsomere 5 with a small and blunt internomedial protuberance. Protarsal claws strongly bent, unsplit at apices, inner one distinctly larger than the outer one. Empodium with one long seta. Protibial notch distinct. Dorsal surface of protibia with dense, erect short setae at inner half.

***Middle and hind legs*.** Yellowish brown; joints of trochanters, femora, and tibiae (base) pale reddish brown; apex of tibial spurs, as well as tarsi and claws reddish brown. Mesotibia with two sharp teeth at apex, the upper one smaller. Metatibia with an apically subtruncate ramus at apex, the ridge with several small teeth. Base of the sharply protruding ventrolateral apices of tarsomeres 4 with a large spiniform seta at each side; tarsomeres 5 with a large and sharp internobasal protuberance. Outer mesotarsal claws widely and deeply split at apex and forming two branches, upper branches slightly thinner and sharper, that of hindlegs also longer than the lower one; inner tarsal claw simply sharp at apex. Empodium of mesotarsus with one long seta, of metatarsus with two long setae. Femora and inner surface of tibia with dense and very long setae, tibia with dense, semierect, and moderately long setae.

***Male genitalia*.** As Figs 9, 10. Parameres strongly asymmetric, basally fused. Phallobase strongly curved in lateral view.

##### Male paratypes.

Most specimens consistent in morphological features, small-sized male (Fig. 4) has distinctly shorter mandible with proximal tooth absent, pronotum narrower and less convex (1.41 × as wide as long).

##### Female paratypes

(mainly based on individual of Figs 5, 6, with modification based on variability of paratype series). ***General*.** Body more elongated ovoid than male, coloration similar to male.

***Head*.** Clypeus flat, subtrapezoidal (posterior margin ca. 3 × wider than anterior margin), anterior margin weakly sinuate and distinctly reflexed, anterior corner broadly rounded, side strongly convergent anteriad and weakly swollen in basal two fifth, then roundly curved, and strongly convergent anteriad to anterior corner; punctures large and almost not spaced. Frontal-clypeal suture formed by a sinuate row of large and not spaced punctures. Frons and vertex with irregular large punctures in anterior half, punctures smaller at inner side of eye. Mandible reddish brown, short, anterior edge strongly reflected with two apically blunt teeth, outer edge weakly concave medially and convex basally. Anterior margin of mentum distinctly bilobed. Antennomeres 3–7 somewhat abbreviated.

***Pronotum*.** Pronotum less convex than male, ca. 1.51 ×as wide as long, anterior margin strongly bisinuate; sides feebly convergent anteriad in posterior half, roundly and broadly curved at middle, then strongly convergent anteriad and feebly concave in anterior half; anterior angle distinctly protruding, posterior angle round.

***Scutellum*.** Scutellum more triangular than male.

***Elytra*.** Elytral surface less uneven compared to male, with irregular and vague longitudinal costae; lateral portion of elytron distinctly bulging behind midpoint; punctures sparser.

***Pygidium*.** Pygidium not strongly convex, subtriangular, not bent to ventrum; setae denser than in male.

***Abdominal ventrites*.** Abdominal ventrites light yellowish brown, slightly darkened at posterior margin of each ventrite; ventrites 2–4 not abbreviated, ventrite 6 not concave at apex; extensively bearing moderately dense punctures, ventrite 6 broadly rugopunctate medially.

***Legs*.** Procoxae situated closer. Three teeth of protibia almost equal in size and shape, not very sharp at apices. Protarsus not thickened, base of the sharply protruding ventrolateral apices of protarsomere 4 with a large spiniform seta at each side, protarsomere 5 without internomedial protuberance. Protarsal claws less bent than in male, two claws almost equal in size; the inner protarsal claw widely and deeply split into two branches, the lower branch is a small dent at middle of inner protarsal claw. Empodium of protarsus with two long setae. Dorsal surface of protibia with sparser setae formed in rows. Metafemur thicker than in male. Protibia and mesotibia feebly curved inward.

##### Measurements.

Body length from apex of clypeus to apex of elytron: 15.6–18.3 mm of male (holotype 17.1 mm) and 15.9–18.1 mm of female; greatest width: 9.2–10.6 mm of male (holotype 9.9 mm) and 8.9–10.0 mm of female.

##### Differential diagnosis.

The new species is most similar to Laotian *D.ohbayashii* (Nagai, 2004), but the large-sized male of the new species has distinctly shorter mandibles. The large and acute basal protrusion is absent in upper margin of mandible in male of *D.ohbayashii*. The parameres of the two species are basally fused and strongly asymmetric. However, the parameres of *D.tangzhaoyangi* differ as follows: the left paramere without an incision at outer margin, apex of the left paramere distinctly narrower, the right paramere strongly curved outward proximally (orients apically in *D.ohbayashii*). The female of *D.ohbayashii* has more concave outer edge of mandible.

##### Etymology.

The specific epithet is dedicated to Zhao-Yang Tang, who generously provided most of the type material of the new taxon.

##### Distribution.

China (Yunnan: Dehong Prefecture).

#### ﻿ Didrepanephorus birmanicus

Taxon classificationAnimaliaColeopteraScarabaeidae

(Arrow, 1907) comb. nov.

17B31633-B794-5791-930A-48C3A0263F07

Figs 8 , 11–15

Fruhstorferia birmanica Arrow, 1907: 354 [original description]; Arrow 1917: 49 [partim], fig. 15; Ohaus 1918: 43 [catalogued]; Ohaus 1934b: 121 [catalogued]; Machatschke 1972: 53 [catalogued]; Muramoto and Araya 2000: 12, figs 3–4 [syntype male figured]; Krajčík 2007: 70 [catalogued]; Krajčík 2012: 110 [catalogued, subgeneric placement unnoted].
Fruhstorferia (Kibakoganea) birmanica Arrow, 1907: Nagai 1984: 29 [catalogued].

##### Type locality.

“Burma, Ruby Mines”, in currently Mogok City of Mandalay, Myanmar.

##### Type material

(1 specimen). ***Lectotype*** of *Fruhstorferiabirmanica* Arrow, 1907 (hereby designated). ♂ (BMNH), “Birmah [R]uby M^es^ // Doherty // 64607 // Fry Coll. 1905-100. // *Fruhstorferiabirmanica*, Arrow type ♂ A. a M., 1907. // Type // HOLOTYPE *Fruhstorferiabirmanica* A. M. SOULA det 1994 // NHMUK014379787”. It will be provided with an additional red label: LECTOTYPE *Fruhstorferiabirmanica* Arrow, 1907 des. Zhao Ming-Zhi 2021.

##### Remarks.

The taxon *Fruhstorferiabirmanica* was originally described based on a pair of specimens from Ruby Mines, Burma (Arrow 1907). The lectotype has not yet been designated and the male illustrated by Muramoto and Araya (2000) has been erroneously fixed as holotype. According to Art. 74.7.1 (ICZN 1999), both specimens should be regarded as syntypes. The examination of both syntypes reveals that the female is conspecific with *D.mizunumai* Nagai & Hirasawa, 1991. Therefore, a lectotype designation is necessary.

**Figures 11–18.**
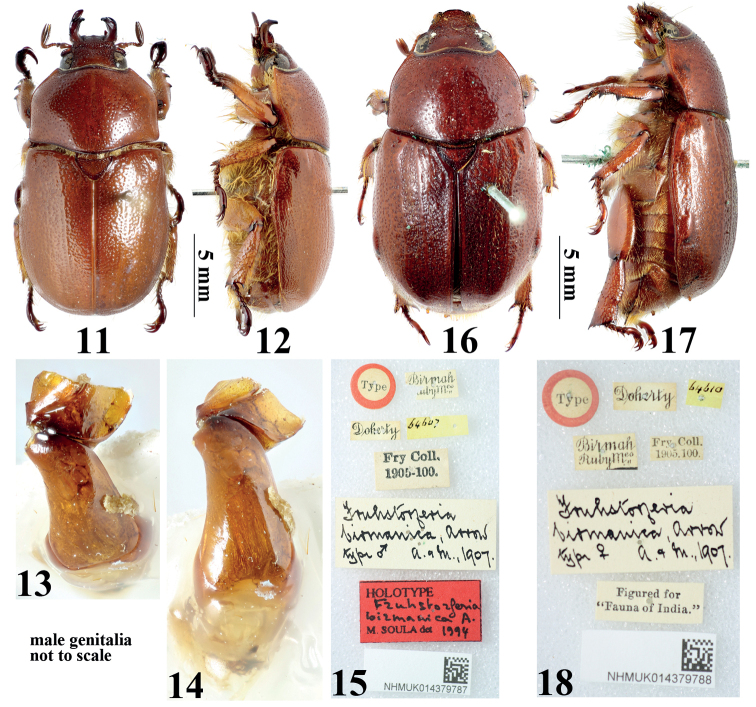
Type material of *Didrepanephorusspecies*. **11–15** male of *D.birmanicus* (Arrow, 1907) (Lectotype of *Fruhstorferiabirmanica* Arrow, 1907) **16–18** female of *D.mizunumai* Nagai & Hirasawa, 1991 (paralectotype of *F.birmanica*), habitus in dorsal view **11, 16** habitus in dorsal view **12, 17** habitus in lateral view **13** aedeagus showing parameres in dorsal view **14** aedeagus in dorsal view **15, 18** attached labels. All photos © Keita Matsumoto (NHMUK).

The male genitalia of the lectotype (Figs 8, 11–15) is partly damaged, which greatly complicates the comparison to its related species. However, the similarity of this species to small-sized male of *D.yunnanus* (Ohaus, 1911) is still apparent in many external features, especially in the allied shape of terminal segment of maxillary palp, which is more expanded and compressed than other *Didrepanephorus*species. There is a strong concavity at the base of lower margin of mandible. The protrusion at lower margin is partly hidden under the labrum. These characters match the above definitions for *Didrepanephorus*. Thus, this species is transferred to the genus *Didrepanephorus* herein. Among the five subspecies of *D.yunnanus*, i.e., *D.y.yunnanus* (Ohaus, 1911), *D.y.clermonti* Benderitter, 1929, *D.y.piaoacensis* Nagai, 2004, *D.y.wakaharai* Nagai, 2004, and *D.y.kachinensis* Muramoto, 2005, recognized by Muramoto (2005), *D.y.kachinensis* appears to be most similar to *D.birmanicus* due to the generally reddish brown body in combination to the closest geographical distance. To fully understand the relation between the two species, examination of topotypical specimens of *D.birmanicus* with undamaged male genitalia is needed.

This species was partly misinterpreted and the name was previously applied to two different species (see below). This species is so far only known from the lectotype.

##### Distribution.

Myanmar (Mandalay Region: Mogok).

#### ﻿ Didrepanephorus fukinukii

Taxon classificationAnimaliaColeopteraScarabaeidae

(Muramoto & Araya, 2000) comb. nov.

544F6868-4A5D-56B4-AD29-9F5109177EF0

Fruhstorferia fukinukii Muramoto & Araya, 2000: 12, figs 1–2, 7 [original description]; Nagai 2004: 150, figs 21–22, 30 [additional record from Mt. Doi Suthep, near Chiang Mai]; Krajčík 2007: 71 [catalogued]; Krajčík 2012: 110 [catalogued, subgeneric placement unnoted].
Fruhstorferia birmanica Arrow, 1907: Nagai and Hirasawa 1991: 7, figs 19, 29–31 [recorded from Northwest Thailand]; Young 1999: 357, fig. 1b; Nagai 2004: 150, figs 19–20, 29 [recorded from Thailand, Fang].

##### Type locality.

North Thailand, Chiang Mai Province, Fang.

##### Material examined

**(2 specimens).** 1♂, 1♀ (GGPC), Thailand, Chiang Mai, Fang, 07.2015.

##### Remarks.

This species is transferred to the genus *Didrepanephorus* here because of the distinct basal protrusion at the lower margin of mandible, as shown by Nagai and Hirasawa (1991).

Judging from the figures of habitus and male genitalia, the records of *F.birmanica* from Northwest Thailand (Nagai and Hirasawa 1991; Nagai 2004) are considered as *D.fukinukii* here. Thus, the record of *F.birmanica* in Thailand should be omitted. Arrow (1917, 1919) and Muramoto and Araya (2000) partly misinterpreted the taxon *F.birmanica* and applied the name to a species from Chin Hills. The species from Chin Hills appears to be very similar to *D.fukinukii* but having different male genitalia, which has strong incision at outer margin of left paramere.

##### Distribution.

Thailand (Chiang Mai).

#### ﻿ Didrepanephorus mizunumai

Taxon classificationAnimaliaColeopteraScarabaeidae

Nagai & Hirasawa, 1991

DF09F378-981E-5386-8D55-E4A895CD8DFD

Figs 16–18

Fruhstorferia birmanica Arrow, 1907: Arrow 1907: 354 [partim, female]; Arrow 1917: 49, fig. 16 [partim, female].
Didrepanephorus mizunumai Nagai & Hirasawa, 1991: 10, figs 2–5, 20, 32–34, 39–40 [original description]; Nagai 2005: 271, figs 4–6, 17; Muramoto 2009: 59, figs 4–6.
Fruhstorferia mizunumai (Nagai & Hirasawa, 1991): Jameson 1997: 170, figs 21–22 [new combination]; Nagai 2004: 150, figs 13–16, 27 [recorded from Houa Phan Prov., Laos]; Krajčík 2007: 71 [catalogued]; Krajčík 2012: 93 [catalogued, subgeneric placement unnoted].
Fruhstorferia yunnana Ohaus, 1911: Muramoto 1993: 7, figs 6, 10 [misidentification, recorded from Sapa, N. Vietnam].

##### Type locality.

North Thailand, Chiang Mai Province, Fang.

##### Material examined

(5 specimens). 1♀ (BMNH), “Birmah Ruby M^es^ // Doherty // 64610 // Fry Coll. 1905. 100. // *Fruhstorferiabirmanica*, Arrow type ♀ A. a M., 1907. // Figured for “Fauna of India.” // Type // NHMUK014379788”, it will be provided with an additional red label: PARALECTOTYPE *Fruhstorferiabirmanica* Arrow, 1907 des. Zhao Ming-Zhi 2021; 1♂, 1♀ (ZMPC), Thailand, Chiang Mai, Fang, 26.VI.2011; 1♂, 1♀ (ZMPC), same as preceding but 2015.V.

##### Remarks.

The female of *D.mizunumai* is easily characterized by the three strongly elevated costae between humeral and apical umbone, as well as a bulge on lateral portions of elytron. The paralectotype of *F.birmanica* (Figs 16–18) well fits topotypical female of *D.mizunumai* from Fang. Therefore, the female paralectotype represents a new distributional record for Myanmar. Two similar species, i.e., Laotian *D.lao* Nagai, 2005 and Vietnamese *D.zen* Muramoto, 2009 have minor morphological differences, but both are more restricted in their known distribution ranges.

##### Distribution.

Laos (Houaphanh); Myanmar (Mandalay) (new record); Thailand (Chiang Mai); Vietnam (Lao Cai).

## ﻿Discussion

In this study, we propose a new delimitation for *Didrepanephorus* and *Fruhstorferia*, which temporarily solves those taxonomic conflicts at generic level. But the placement of *D.takuyai* (Muramoto, 2003) remains uncertain and this species requires further examination. Type material of five of the seven taxa of the genus *Didrepanephorus* described before 2000 had been re-examined and illustrated (Muramoto and Fujioka 2000; Muramoto 2005; Qiu et al. 2021; the present paper). Species described in this century were all well-illustrated. Hence there is a good taxonomic fundament at specific or infraspecific level. To date, the genus *Didrepanephorus* comprises 21 valid taxa distributed in the Indochina Peninsula, southern China, and the Himalaya. Two thirds of the valid taxa were described in this century and it is likely that new species will be discovered in the future. The Chin Hills species similar to *D.fukinukii* should be studied to ensure its status. The registered Chinese fauna of *Didrepanephorus* increased rapidly from two to six species within two years. New faunistic records can be expected due to the existence of other congeners known from the adjacent regions.

Moreover, no phylogenetic analysis was conducted for *Didrepanephorus* and related genera, i.e., *Fruhstorferia*, *Kibakoganea*, *Masumotokoganea*, *Nagainokoganea* and *Pukupuku*. In the morphology-based phylogenetic study (Jameson 1997), *D.mizunumai* and *K.sexmaculata* were used as representatives of the subtribe Fruhstorferiina Ohaus, 1918 and formed a clade together with the genera *Ceroplophana* Gestro, 1893, *Dicaulocephalus* Gestro, 1888 and *Peperonota* Westwood, 1847, which were traditionally recognized as members of the subtribe Parastasiina Burmeister, 1844 (Ohaus 1918, 1934b). It suggests that these three genera should be taken into consideration as well.

## Supplementary Material

XML Treatment for Didrepanephorus tangzhaoyangi

XML Treatment for Didrepanephorus birmanicus

XML Treatment for Didrepanephorus fukinukii

XML Treatment for Didrepanephorus mizunumai
